# Investigation of temperature rise characteristics in the pre-stage of a deflector jet servo valve

**DOI:** 10.1038/s41598-025-21819-4

**Published:** 2025-10-30

**Authors:** Li Ma, Lianhao Wan, Yin Liu, Jianbin Wang, Hao Yan

**Affiliations:** 1 School of Mechanical and Automotive Engineering, Anhui Polytechnic University, Wuhu, 241000 China; 2 School of Mechanical, Electronic and Control Engineering, Beijing Jiaotong University, Beijing, 100044 China; 3 Key Laboratory of Vehicle Advanced Manufacturing, Measuring and Control Technology, Beijing Jiaotong University, Ministry of Education, Beijing, 100044 China; 4 Beijing Jiaotong University, No.3 Shangyuancun Haidian District, Beijing, 100044 PR China

**Keywords:** Deflector jet servo valve, Temperature variation, Thermodynamic model, Engineering, Physics

## Abstract

In aerospace equipment, the deflector jet servo valve is a high-end core control component of electro-hydraulic servo systems. The pre-stage is crucial because of the compact structure and complex jet morphology. Temperature variations can significantly impair the valve’s control accuracy. To address the mechanism of heat generation and temperature prediction inside the pre-stage, a thermodynamic model of the pre-stage is developed using the control volume method to predict heat generation and temperature rise under varying operating conditions. This model enables prediction of temperature distribution across various chambers and the casing based on specified deflector displacements. A dedicated temperature-controlled test rig is established to measure the temperature distribution on the servo valve casing. Comparison of simulation results with experimental data validated the accuracy of the proposed model, and the temperature distribution predicted by the model has less than 0.76 °C error compared to the experiment. This study provides a theoretical basis for performance enhancement and structural optimization of deflector jet servo valves.

## Introduction

As a high-end core control component of hydraulic servo systems, the electro-hydraulic servo valve is known for the high power transmission, rapid response, and precise control. However, high temperatures can lead to oxidative decomposition and degradation of hydraulic fluid, reduced viscosity, accelerated seal aging, valve sticking, and poor lubrication of seals^1^.

Existing research on the impact of temperature variations on deflector jet servo valves has primarily focused on the structural aspects of the torque motor and spool valve, while a comprehensive investigation into the influence of the pre-stage is still lacking^2^. A numerical simulation study by Zhao et al.^3^ on the three-dimensional thermal-fluid-structure coupling of a specific type of servo valve under extreme operating conditions revealed its heat transfer path and dispersion mechanism. Chen and Wang^4^ applied the control variable method to categorize oil viscosity at different temperatures. Zhang et al.^5^ developed a mathematical model for the internal flow field of the electro- hydraulic servo valve under temperature variations. An optimization algorithm (M-OA) for valve orifice thermal deformation was proposed by Chen et al.^6^ to address the issue of valve spool sticking caused by thermal deformation. Simulation results included temperature rise and thermal deformation data of the orifice, while the clamping force of the valve spool at different temperatures was measured using a temperature control method^7^. Finite element methods were employed by Zhao et al.^8^ for thermal-fluid-solid coupling numerical simulations of the valve spool, detailing the temperature and thermal deformation distribution in the casing, valve spool, and valve sleeve, as well as the pressure distribution, velocity distribution, and temperature field of the flow passage. Mao^9^ refined the flow field variation pattern under temperature shock by integrating jet flow with thermodynamics theory. Sun^10^ investigated the thermal deformation of pre-stage structures such as the jet pan and deflector across wide temperature ranges.

The modeling and simulation of thermal characteristics in hydraulic systems often involve several methods, including the power loss, nodal, control volume, computational fluid dynamics (CFD), and neural network methods^11^. The concept of control volumes was proposed by Sidders et al.^12^, and mathematical models were established to analyze the energy exchange processes of hydraulic components. Li^13^ developed a basic model for hydraulic components using the control volume method and established a parameter mathematical model based on energy conservation. Simplification methods for differential equations were also suggested to reduce computation time^14,15^.

The thermal characteristic model for hydraulic motors was first established by Li and Jiao^16^ using the control volume method. Subsequently, thermodynamic models for spool valves^17^, hydraulic cone valves^18^, and plunger pumps^19^ were developed, taking into consideration the principles of thermal characteristic modeling. Kim et al.^20^ estimated the pressure loss of servo valves using computational fluid dynamics results and validated the accuracy of the thermal characteristic analysis through both simulation and experimental results. Zhou^21^ and Zhao et al.^22^ performed finite element thermal-flow-solid coupling simulations for nozzle flapper servo valve.

Based on the current research status, it is evident that studies on the deflector jet servo valve at extreme temperatures primarily focus on numerical simulations of the valve spool, the impact of temperature on the torque motor structure, and thermal modeling of hydraulic systems. However, comprehensive theoretical investigations into the effects of temperature variations on the pre-stage of the deflector jet servo valve remain lacking.

This study establishes a thermodynamic model for pre-stage coupled jet dynamics and shell heat transfer which simplifies the pre-stage of the deflector jet servo valve using volumetric and damped control volume equations. Numerical solutions are performed using specified signals to predict temperature rise in various chambers and the shell of the servo valve. A dedicated test rig for evaluating the temperature characteristics of the deflector jet servo valve is constructed. Temperature distribution of the valve shell are obtained using thermocouples along with thermal imaging devices, thereby validating the accuracy of the proposed thermodynamic model.

## Thermodynamic model of the pre-stage of the deflector jet servo valve

### Theoretical foundation of the pre-stage of the deflector jet servo valve

**Fig. 1:**
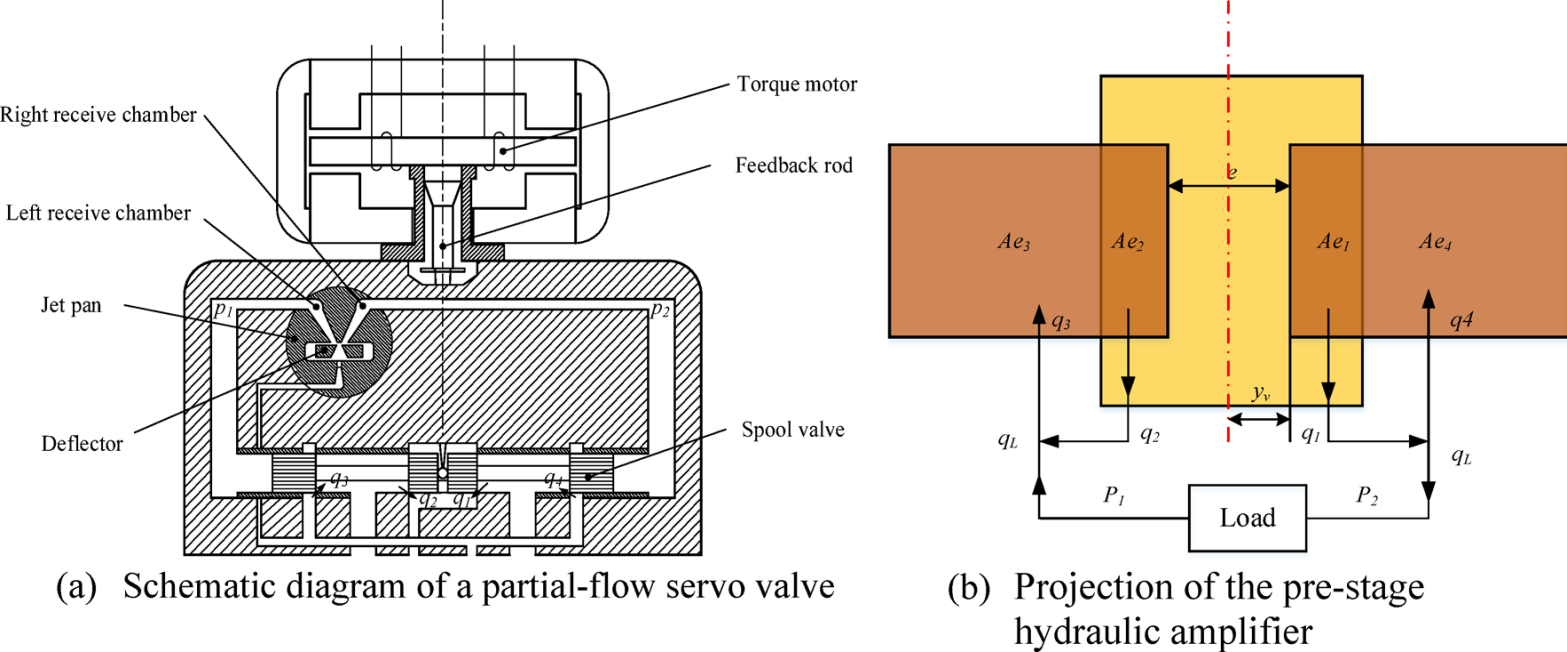
Schematic diagram and projection of the deflector jet servo valve.

The control volume method, grounded in the first law of thermodynamics, is utilized for the thermodynamic modeling and analysis of hydraulic components. Based on this approach, models for temperature and pressure variations are derived to describe temperature and pressure variations within the control volume^23^. Although the pre-stage of the deflector jet servo valve is not operated according to the throttling principle, it is still analyzed and computed using throttling theory. The projection of the hydraulic amplifier for the pre-stage of the deflector jet servo valve is illustrated in Fig. 1., ${A_{e2}}$, and ${A_{e4}}$ is the flow area, ${q_1}$, ${q_2}$, ${q_3}$ and ${q_4}$ is the corresponding flow rate, $e$ is the distance between the left and right receiving cavities, and ${y_v}$ is the offset of the deflecting plate. The flow rates of the four outlets are as follows.

1 $${q_1}=w_{j}^{\prime }\left( {\frac{{{\lambda _d}w_{j}^{\prime } - w_{e}^{\prime }}}{2}+{y_v}} \right){C_{csj}}\sqrt {\frac{2}{\rho }({P_{csj}}\cos {\theta _r} - {P_1})}$$

2 $${q_2}=w_{j}^{\prime }\left( {w_{r}^{\prime } - \frac{{{\lambda _d}w_{j}^{\prime } - w_{e}^{\prime }}}{2} - {y_v}} \right){C_{cd}}\sqrt {\frac{2}{\rho }({P_1} - {P_r})}$$

3 $${q_3}={w_j}\left( {w_{r}^{\prime } - \frac{{{\lambda _d}w_{j}^{\prime } - w_{e}^{\prime }}}{2}+{y_v}} \right){C_{cd}}\sqrt {\frac{2}{\rho }({P_2} - {P_r})}$$

4 $${q_4}=w_{j}^{\prime }\left( {\frac{{{\lambda _d}w_{j}^{\prime } - w_{e}^{\prime }}}{2} - {y_v}} \right){C_{csj}}\sqrt {\frac{2}{\rho }({P_{csj}}\cos {\theta _r} - {P_2})}$$

According to Fig. 1 and Eqs. 1–4, the leakage flow in the pre-stage hydraulic amplifier of the deflector jet servo valve is as follows.

5 $${q_c}=[{w_j}{w_h} - {A_{e1}}(x) - {A_{e2}}(x)]{C_{sj}}\sqrt {\frac{2}{\rho }{P_s}}$$

**Fig. 2:**
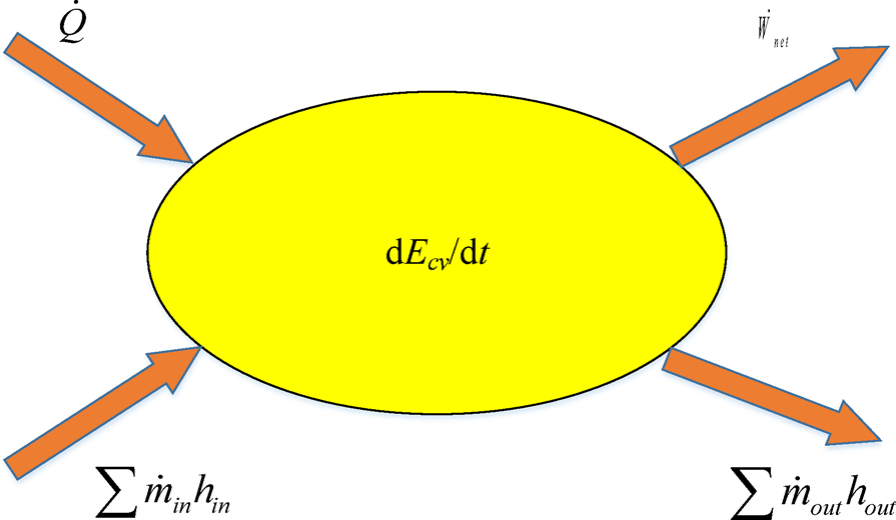
Internal volumetric hydraulic component of the servo valve.

For the deflector jet servo valve, in the modeling process, the spool valve is set as a load. Therefore, the internal chambers of the servo valve are divided into two types: volumetric and damping. The pressure and temperature in each part of the volumetric control body tend to be the same, and there is no obvious pressure and temperature gradient in space. The pressure transformation depends on the fluid state in and out of the control body and the volume change of the control body^23,24^. The thermodynamic system of the volumetric hydraulic component is shown in Fig. 2.

The internal control body was considered the research object, the heat exchange between the system and outside world is $\dot {Q}$, the external work on the system is ${\dot {W}_s}$, the energy contained in the oil flowing into the control body is$\sum {{{\dot {m}}_{in}}} {h_{in}}$, and the energy contained in the heating oil of the outflow control body is $\sum {{{\dot {m}}_{out}}} {h_{out}}$. Assuming that the kinetic energy and potential energy are not considered in the oil flow process, the energy equation of the control body is

6 $$\frac{{{\text{d}}{E_{cv}}}}{{{\text{d}}t}}=\dot {Q}+{\dot {W}_s}+{\dot {W}_b}+\sum {{{\dot {m}}_{in}}} {h_{in}} - \sum {{{\dot {m}}_{out}}} {h_{out}}$$

where ${h_{in}}$ and ${h_{out}}$ are the enthalpy values of oil at the input and output ends, respectively, and ${E_{cv}}$ is the internal energy controlling the oil in the body.

For $$\frac{{{\text{d}}{E_{cv}}}}{{{\text{d}}t}}$$,

7 $$\frac{{{\text{d}}{E_{cv}}}}{{{\text{d}}t}}=\frac{{{\text{d}}(mu)}}{{{\text{d}}t}}=m\frac{{{\text{d}}u}}{{{\text{d}}t}}+u\frac{{{\text{d}}m}}{{{\text{d}}t}}$$

The second enthalpy equation for the flow is denoted by

8 $${\text{d}}h={c_p}{\text{d}}T+(1 - {\alpha _p}T)v{\text{d}}p$$

Applying the second enthalpy equation, Eq. (7) can be expressed as

9 $$\frac{{{\text{d}}{E_{cv}}}}{{{\text{d}}t}}=m{c_p}\frac{{{\text{d}}T}}{{{\text{d}}t}} - {\alpha _p}Tmv\frac{{{\text{d}}p}}{{{\text{d}}t}}+h\frac{{{\text{d}}m}}{{{\text{d}}t}} - p\frac{{{\text{d}}V}}{{{\text{d}}t}}$$

where $\frac{{{\text{d}}V}}{{{\text{d}}t}}$ is the rate of change controlling the volume of the body, $\frac{{{\text{d}}m}}{{{\text{d}}t}}$ is to control the rate of body mass change.

The change rate of oil mass in the control body is given by

10 $$\frac{{{\text{d}}m}}{{{\text{d}}t}}=\sum {{{\dot {m}}_{in}}} - \sum {{{\dot {m}}_{out}}}$$

Substituting Eqs. (9) and (10) into Eq. (6), we obtain the following:

11 $$\frac{{{\text{d}}T}}{{{\text{d}}t}}=\frac{1}{{m{c_p}}}[\sum {{{\dot {m}}_{in}}({h_{in}} - h)} +\sum {{{\dot {m}}_{out}}(h - {h_{out}})} +\dot {Q}+{\dot {W}_s}+{\dot {W}_b}+p\frac{{{\text{d}}V}}{{{\text{d}}t}}+{\alpha _p}Tm\nu \frac{{{\text{d}}p}}{{{\text{d}}t}}]$$

where the boundary work can be expressed as

12 $${W_b}= - p{\text{d}}V$$

If the direction of fluid flow remains constant, it can be considered $h={h_{out}}$. Meanwhile, for the thermodynamic analysis of the servo valve, the influence of shaft work can be ignored (exactly ${\dot {W}_s}=0$). Taking the time derivative of Eq. (12) and substituting it into Eq. (11) gives

13 $$\frac{{{\text{d}}T}}{{{\text{d}}t}}=\frac{1}{{m{c_p}}}[\sum {{{\dot {m}}_{in}}({h_{in}} - h)} +\dot {Q}+{\alpha _p}Tm\nu \frac{{{\text{d}}p}}{{{\text{d}}t}}]$$

A simplified enthalpy expression can be obtained from the second low of enthalpy.

14 $${h_{in}} - h={\bar {c}_p}({T_{in}} - T)+(1 - {\bar {c}_p}\bar {T})\bar {\nu }({P_{in}} - P)$$

where $\overline {{{c_p}}} ={c_p}(\overline {p} ,\overline {T} )$ is the average specific heat capacity of the control body, $\overline {p} ={{({P_{in}}+P)} \mathord{\left/ {\vphantom {{({P_{in}}+P)} 2}} \right. \kern-0pt} 2}$ and $\overline {T} ={{({T_{in}}+T)} \mathord{\left/ {\vphantom {{({T_{in}}+T)} 2}} \right. \kern-0pt} 2}$ represent the average pressure and temperature in the body, respectively.

Equation (14) serves as the dynamic temperature equation for the volumetric control volume within the deflector jet servo valve. The rate of temperature change within the control volume is associated with the differences in enthalpy values of the fluid entering and within the control volume, as well as with heat exchange and pressure rate changes.

**Fig. 3:**
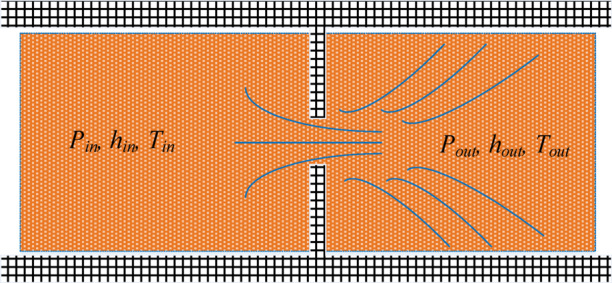
Internal damping element of the servo valve.

In the damping-type control volume, significant pressure gradients exist across the throttling orifices. The loss of pressure energy is converted into viscous dissipation heat in the oil, making it unsuitable to describe the control volume state solely based on pressure and temperature. Under steady-state conditions, the flow rate through the damping element remains constant. The flow rate through the control volume of the damping-type element within the servo valve can be determined using the throttling equations from Eq. (1) to (4). The thermodynamic system of the damping-type hydraulic component is illustrated in Fig. 3. Because the throttling process of the damping element is minimal, no shaft work or volumetric work exists in the control volume. The kinetic and potential energies and heat exchanges with the external environment during fluid ingress and egress are neglected. The energy contained in the oil before and after the throttling orifice remains unchanged, and the enthalpy values of the fluid entering and exiting the throttling orifice are constant.

15 $${h_{in}}={h_{out}}$$

By substituting Eq. (15) into Eq. (14), we obtain the following Equation.

16 $$\left\{ \begin{gathered} 0={{\bar {c}}_p}({T_{in}} - {T_{out}})+(1 - {{\bar {c}}_p}\bar {T})\bar {\nu }({P_{in}} - {P_{out}}) \hfill \\ \bar {T}=({T_{in}}+{T_{out}})/2 \hfill \\ \end{gathered} \right.$$

Equation (16) shows that when the fluid flows through the damping-type element, all the lost pressure energy is converted into the internal energy of the fluid, indicating that the temperature difference in the fluid across the damping-type element is determined solely by the pressure difference.

### Thermodynamic model of the deflector jet servo valve

**Fig. 4:**
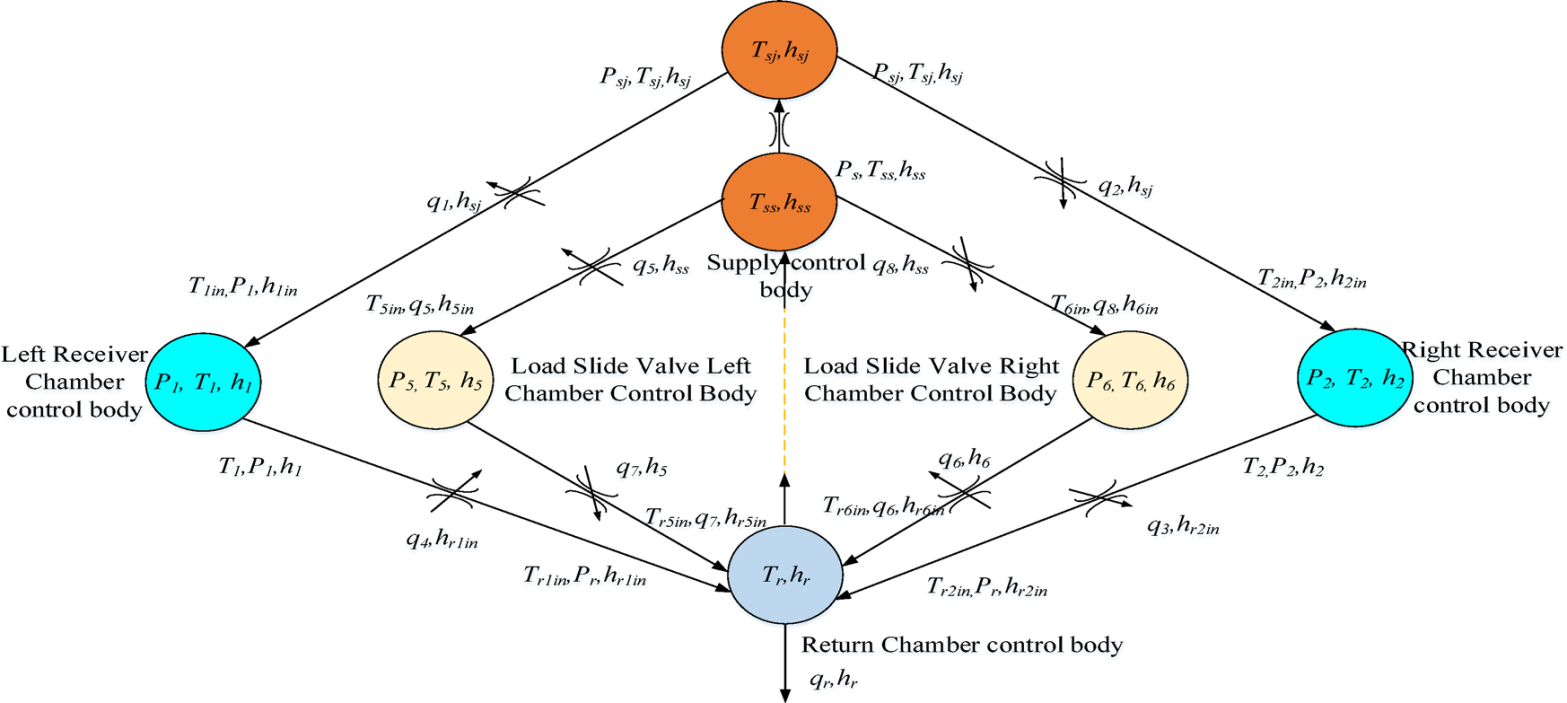
Control volume model of the deflector jet servo valve.

When the fluid flows within the deflector jet servo valve, the spool valve is set as the load, and the process of fluid flowing through the internal channels is simplified to the fluid passing through multiple volumetric and damping-type control elements. The internal oil circuit diagram with the servo valve in the neutral position and selection of control elements are represented as shown in Fig. 4. The inlet and return channels are designated as volumetric control elements, while the jet nozzle, left and right receiver chambers, and the rectangular throttling orifices of the spool valves are designated as damping-type control elements. Additionally, since the movement speed of the valve spool within the valve sleeve is less, the heat generated by its movement is neglected in the modeling process. The heat produced by the current in the torque motor coil is minimal, and the resultant temperature increase is not considered in the rise of fluid temperature. Consequently, the thermodynamic model is simplified by not considering the torque motor heating and by assuming the shell conductivity.

The pre-stage control element model of the deflector jet servo valve is analogous to a hydraulic equivalent bridge circuit, where all four chambers are variable throttling orifices. Considering that the internal and inlet/outlet temperatures and pressures of the supply control elements remain constant, their equations can be omitted. According to Eq. (16), the temperature equations for the initial jet nozzle control element and the left and right receiver chamber control elements are represented as follows.

17 $$\left\{ {\begin{array}{*{20}{c}} {\frac{{{\text{d}}{T_{sj}}}}{{{\text{d}}t}}=\frac{1}{{{c_p}{m_{sj}}}}[{q_s}\rho ({h_{ss}} - {h_{sj}}) - {{\dot {Q}}_{sj}}]+\frac{{{\alpha _p}{T_{sj}}}}{{{c_p}\rho }}\frac{{{\text{d}}{P_{sj}}}}{{{\text{d}}t}}} \\ {\frac{{{\text{d}}{T_1}}}{{{\text{d}}t}}=\frac{1}{{{c_p}{m_1}}}[{q_1}\rho ({h_{1in}} - {h_1}) - {{\dot {Q}}_1}]+\frac{{{\alpha _p}{T_1}}}{{{c_p}\rho }}\frac{{{\text{d}}{P_1}}}{{{\text{d}}t}}} \\ {\frac{{{\text{d}}{T_2}}}{{{\text{d}}t}}=\frac{1}{{{c_p}{m_2}}}[{q_2}\rho ({h_{2in}} - {h_2}) - {{\dot {Q}}_2}]+\frac{{{\alpha _p}{T_2}}}{{{c_p}\rho }}\frac{{{\text{d}}{P_2}}}{{{\text{d}}t}}} \end{array}} \right.$$

where ${T_{ss}}$, ${T_{sj}}$, ${T_1}$, and ${T_2}$ are the temperatures of the oil supply, jet nozzle, and left and right receiver chambers, respectively; ${m_{sj}}$, ${m_1}$, and ${m_2}$ are the control body masses of the jet nozzle and left and right receiver chambers, respectively; ${h_{ss}}$, ${h_1}$, and ${h_2}$are the oil supply, jet nozzle, and left and right receiver chambers controlling the enthalpy value of the oil, respectively; ${h_{1in}}$ and ${h_{2in}}$ are the oil enthalpy values at the inlet of the left and right receiver chambers, respectively; and ${\dot {Q}_{sj}}$, ${\dot {Q}_1}$, and ${\dot {Q}_2}$ are the heat flow values between the jet nozzle and left and right receiver chambers and the shell of the deflector jet servo valve, respectively.

According to Eq. (14), the change in enthalpy in Eq. (17) is denoted by

18 $$\left\{ \begin{gathered} {h_{ss}} - {h_{sj}}={c_p}({T_{ss}} - {T_{sj}}) \hfill \\ {h_{1in}} - {h_1}={c_p}({T_{1in}} - {T_1}) \hfill \\ {h_{2in}} - {h_2}={c_p}({T_{2in}} - {T_2}) \hfill \\ \end{gathered} \right.$$

The deflector jet control body model is similar to the hydraulic equivalent bridge, where all four chambers function as variable throttles, and the pressure loss before and after the throttles is transformed into the internal energy of the oil. According to Eq. (16), the temperature calculation equation of the four throttles is

19 $$\left\{ \begin{gathered} 0={c_p}({T_{ss}} - {T_{sjin}})+(1 - {\alpha _p}({T_j}+{T_{sjin}})/2)({P_s} - {P_{sj}})/\rho \hfill \\ 0={c_p}({T_{sj}} - {T_{1in}})+(1 - {\alpha _p}({T_{sj}}+{T_{1in}})/2)({P_{sj}} - {P_1})/\rho \hfill \\ 0={c_p}({T_{sj}} - {T_{2in}})+(1 - {\alpha _p}({T_{sj}}+{T_{2in}})/2)({P_{sj}} - {P_2})/\rho \hfill \\ 0={c_p}({T_{sj}} - {T_{r1in}})+(1 - {\alpha _p}({T_{sj}}+{T_{r1in}})/2)({P_{sj}} - {P_r})/\rho \hfill \\ 0={c_p}({T_{sj}} - {T_{r2in}})+(1 - {\alpha _p}({T_{sj}}+{T_{r2in}})/2)({P_{sj}} - {P_r})/\rho \hfill \\ \end{gathered} \right.$$

where ${T_{rsjin}}$ is the outlet temperature of the jet nozzle, and ${T_{r1in}}$ and ${T_{r2in}}$ are the oil temperatures at the left and right return ports, respectively.

When the deflector is not in the central position, the kinetic energy of the oil in the left and right receiver chambers of the deflector jet servo valve pre-stage is not equal. Consequently, as the restoring pressures in the left and right load chambers are unequal, a pressure differential is generated across the spool valve, which eventually leads to the movement of valve spool. Subsequently, the direction of oil flow within the left and right control volumes of the spool valve changes, necessitating the establishment of temperature calculation models for two different scenarios.

The hydraulic oil within the spool valve is divided into three volumetric control volumes: the load spool valve left chamber control volume, the load spool valve right chamber control volume, and the return oil chamber control volume. When the valve spool moves to the left (${y_j} \geqslant 0$), a portion of the flow ${q_7}$ from the left chamber control volume enters the return oil control chamber. The flow rate of the leakage entering the right control volume of the spool valve is ${q_8}$. the flow rate of ${q_6}$ through the orifice enters the return oil chamber control volume. When the spool moves to the right (${y_j}<0$), a portion of the flow rate ${q_6}$ from the right control volume of the spool valve leaks into the return oil chamber control volume. The leakage flow in the control body of the left chamber of the spool valve is ${q_5}$. The flow rate ${q_7}$ leaks into the return oil control chamber through the throttle port. The control body pressure, flow rate, and temperature of the left chamber of the spool valve are ${P_1}$, ${q_3}$, and ${T_5}$, respectively. The pressure, flow, and temperature of the control body in the right chamber of the spool valve are ${P_2}$, ${q_4}$, and ${T_6}$,respectively. The control volume pressure, flow rate, and temperature of the oil return chamber are ${P_r}$, ${q_r}$, and ${T_r}$, respectively. The flow rates of the four throttles of the spool are ${q_5}$, ${q_6}$, ${q_7}$, and ${q_8}$. When the valve spool moves to the left, the flow through each flow channel of the spool valve can be expressed as follows^4^.

20 $$\left\{ \begin{gathered} q_{5} = C_{d} wy_{v} \sqrt {\frac{{2(P_{s} - P_{5} )}}{\rho }} + \frac{{wh^{3} (P_{s} - P_{5} )}}{{12uL}} \hfill \\ q_{6} = C_{d} wy_{v} \sqrt {\frac{{2(P_{6} - P_{r} )}}{\rho }} + \frac{{wh^{3} (P_{6} - P_{r} )}}{{12uL}} \hfill \\ q_{7} = \frac{{wh^{3} (P_{5} - P_{r} )}}{{12u(L + y_{v} )}} \hfill \\ q_{8} = \frac{{wh^{3} (P_{s} - P_{6} )}}{{12u(L + y_{v} )}} \hfill \\ \end{gathered} \right.(y_{j} \ge 0)$$

where *h* is fit clearance, *w* is the area gradient, and *L* is the length of the gap.

When the valve spool moves to the right, the flow through each flow channel of the spool valve can be expressed as follows.

21 $$\left\{ \begin{gathered} {q_5}=\frac{{w{h^3}({P_s} - {P_5})}}{{12u(L - {y_v})}} \hfill \\ {q_6}=\frac{{w{h^3}({P_6} - {P_r})}}{{12u(L - {y_v})}} \hfill \\ {q_7}= - {C_d}w{y_v}\sqrt {\frac{{2({P_5} - {P_r})}}{\rho }} +\frac{{w{h^3}({P_5} - {P_r})}}{{12u(L+{y_v})}} \hfill \\ {q_8}= - {C_d}w{y_v}\sqrt {\frac{{2({P_s} - {P_6})}}{\rho }} +\frac{{w{h^3}({P_s} - {P_6})}}{{12u(L+{y_v})}} \hfill \\ \end{gathered} \right.({y_j}<0)$$

According to Eq. (14), when the main valve spool moves to the left, the temperature equation of the control body of the left and right chambers the load spool can be expressed as follows.

22 $$\left\{ \begin{gathered} \frac{{{\text{d}}{T_5}}}{{{\text{d}}t}}=\frac{1}{{{c_p}{m_5}}}[{q_5}\rho ({h_{5in}} - {h_5}) - {{\dot {Q}}_5}]+\frac{{{\alpha _p}{T_5}}}{{{c_p}\rho }}\frac{{{\text{d}}{P_5}}}{{{\text{d}}t}} \hfill \\ \frac{{{\text{d}}{T_6}}}{{{\text{d}}t}}=\frac{1}{{{c_p}{m_6}}}[{q_8}\rho ({h_{6in}} - {h_6}) - {{\dot {Q}}_6}]+\frac{{{\alpha _p}{T_6}}}{{{c_p}\rho }}\frac{{{\text{d}}{P_6}}}{{{\text{d}}t}} \hfill \\ \end{gathered} \right.$$

According to Eq. (11), the corresponding enthalpy change is given by

23 $$\left\{ \begin{gathered} {h_{5in}} - {h_5}={c_p}({T_{5in}} - {T_5}) \hfill \\ {h_{6in}} - {h_6}={c_p}({T_{6in}} - {T_6}) \hfill \\ \end{gathered} \right.$$

where ${m_5}$ and ${m_6}$ are the masses of left and right chambers of the spool valve, respectively, ${h_{5in}}$ and ${h_{6in}}$ are the enthalpy values of the oil at the inlet of the left and right chambers of the spool valve, respectively. ${h_5}$ and ${h_6}$ are the enthalpy values of the control body oil in the left and right chambers of the spool valve, respectively. ${\dot {Q}_5}$ and ${\dot {Q}_6}$ are the heat flows between the left and right chambers of the spool valve and the deflector jet servo valve shell, respectively.

Similarly, when the spool moves to the right,

24 $$\left\{ \begin{gathered} \frac{{{\text{d}}{T_5}}}{{{\text{d}}t}}=\frac{1}{{{c_p}{m_5}}}[{q_5}\rho ({h_{5in}} - {h_5}) - {{\dot {Q}}_5}]+\frac{{{\alpha _p}{T_5}}}{{{c_p}\rho }}\frac{{{\text{d}}{P_5}}}{{{\text{d}}t}} \hfill \\ \frac{{{\text{d}}{T_6}}}{{{\text{d}}t}}=\frac{1}{{{c_p}{m_6}}}[{q_8}\rho ({h_{6in}} - {h_6}) - {{\dot {Q}}_6}]+\frac{{{\alpha _p}{T_6}}}{{{c_p}\rho }}\frac{{{\text{d}}{P_6}}}{{{\text{d}}t}} \hfill \\ \end{gathered} \right.$$

According to Eq. (14), the change in the corresponding enthalpy value is

25 $$\left\{ \begin{gathered} {h_{5in}} - {h_5}={c_p}({T_{5in}} - {T_5}) \hfill \\ {h_{6in}} - {h_6}={c_p}({T_{6in}} - {T_6}) \hfill \\ \end{gathered} \right.$$

The control body of the oil return chamber includes the bidirectional leakage flow from the secondary jet of the deflector and the flow from the bidirectional load chambers of the spool valve or its internal leakage flow. The temperature calculation equation of the control body of the oil return chamber is as follows.

26 $$\frac{{{\text{d}}{T_r}}}{{{\text{d}}t}}=\frac{1}{{{c_p}{m_r}}}[{q_1}\rho ({h_{r1in}} - {h_r})+{q_2}\rho ({h_{r2in}} - {h_r})+{q_6}\rho ({h_{r6in}} - {h_r})+{q_7}\rho ({h_{r5in}} - {h_r}) - {\dot {Q}_r}]+\frac{{{\alpha _p}{T_r}}}{{{c_p}\rho }}\frac{{{\text{d}}{P_r}}}{{{\text{d}}t}}$$

where ${h_{r1in}}$ and ${h_{r2in}}$ are the oil enthalpy values at the left and right oil return ports at the secondary jet flow of the deflector, respectively, ${h_{r5in}}$ and ${h_{r6in}}$ are the enthalpy values of oil flowing into the inlet of the control body of the left and right chambers of the load spool valve, respectively.

The change in the corresponding enthalpy value is denoted by

27 $$\left\{ \begin{gathered} {h_{r5in}} - {h_1}={c_p}({T_{r5in}} - {T_r}) \hfill \\ {h_{r6in}} - {h_2}={c_p}({T_{r6in}} - {T_r}) \hfill \\ {h_{r1in}} - {h_{rn}}={c_p}({T_{r1in}} - {T_r}) \hfill \\ {h_{r2in}} - {h_{rn}}={c_p}({T_{r2in}} - {T_r}) \hfill \\ \end{gathered} \right.$$

where ${T_{r1in}}$ and ${T_{r2in}}$ are the oil enthalpy values at the left and right return oil ports at the deflector jet flow, respectively, ${T_{r5in}}$ and ${T_{r6in}}$ are the enthalpy values of oil flowing into the inlet of the control body of the left and right chambers of the load spool valve, respectively.

For the temperature difference generated by the variable throttle port of the spool valve, according to Eqs. (5–19), the temperature calculation equation of the variable throttle port of the spool valve is as follows.

28 $$\left\{ \begin{gathered} 0={c_p}({T_{ss}} - {T_{5in}})+(1 - {\alpha _p}({T_{ss}}+{T_{5in}})/2)({P_s} - {P_5})/\rho \hfill \\ 0={c_p}({T_{ss}} - {T_{6in}})+(1 - {\alpha _p}({T_{ss}}+{T_{6in}})/2)({P_s} - {P_6})/\rho \hfill \\ 0={c_p}({T_1} - {T_{r5in}})+(1 - {\alpha _p}({T_1}+{T_{r5in}})/2)({P_1} - {P_r})/\rho \hfill \\ 0={c_p}({T_2} - {T_{r6in}})+(1 - {\alpha _p}({T_2}+{T_{r6in}})/2)({P_2} - {P_r})/\rho \hfill \\ \end{gathered} \right.$$

### Thermodynamic model of the shell

**Fig. 5:**
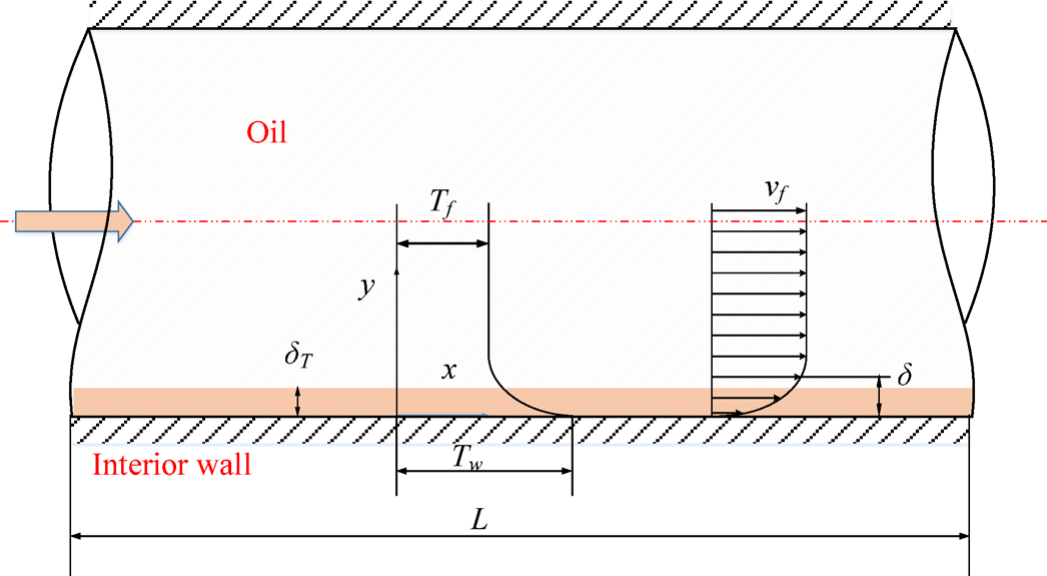
Temperature and velocity boundary layer of the oil in the servo valve flow channel.

Most of the heat energy generated by the throttling of the oil inside the deflector jet servo valve is utilized to increase the temperature of the oil in the return chamber, while work is done on the inner walls of the valve by the oil. The remaining heat is transferred through the shell to the surrounding air by conduction and convection. When the valve reaches a stable thermal state, a steady heat source is present internally, and heat is stably dissipated to the outside. In the heat transfer analysis of the control body and shell within the internal flow channel of the deflector jet servo valve, the forced convection heat transfer coefficient is not constant but varies with changes in the flow conditions. As shown in Fig. 5, a temperature boundary layer is formed on the inner walls as the oil flows through the internal pipes of the deflector jet servo valve^24,25^.

The control body of the internal flow passage of the deflector jet servo valve is simplified into a cylindrical flow passage of forced convection heat transfer. Typically, in the context of engineering, only the convection heat transfer on the entire heat transfer surface of the deflector jet servo valve needs to be considered. The surface convection heat transfer coefficient is determined experimentally based on the similarity principle, and the experimental correlation formula for heat transfer calculations is adopted. For in-tube flow, the surface heat transfer coefficient can be determined according to the Nusselt number:

29 $$h=\frac{{Nuk}}{D}$$

The Nusselt number can be expressed as

30 $$\bar {N}u=\frac{{\bar {h}D}}{k}=\bar {g}({R_e},{P_p})$$

According to Eq. (30), the dimensionless heat transfer coefficient and Reynolds number of forced convective heat transfer are related to the Prandtl number, and the Nusselt number of forced convective heat transfer of pipe flow with a circular section is estimated as 3.66^10^. For a circular cross-section pipeline, the contact area between the inner wall and the oil is

31 $${A_w}=D\pi l$$

where *l* is the pipe length of the inner wall of the flow channel.

By substituting Eqs. (30) and (31) into Eq. (29), the heat flux of the oil and the inner wall of the flow channel can be obtained as

32 $$\dot {Q}=\pi lk\bar {N}u({T_w} - {T_f})$$

Heat is exchanged between the shell of the deflector jet servo valve and the air via natural convection heat transfer, and its heat transfer coefficient is closely related to the temperature field. According to a previous study^21^, the convective heat transfer coefficient between the shell and the air is 20 W/(m^2^K), and the heat flow rate between the shell and the air is

33 $${\dot {Q}_{air}}={A_s}{\bar {h}_s}({T_w} - {T_e})$$

where ${A_s}$ is the contact area between the shell and the air, ${\overline {h} _s}$ is the average convective heat transfer coefficient, ${T_w}$ is the shell temperature, and ${T_e}$ is the air temperature around the deflector jet servo valve.

**Fig. 6:**
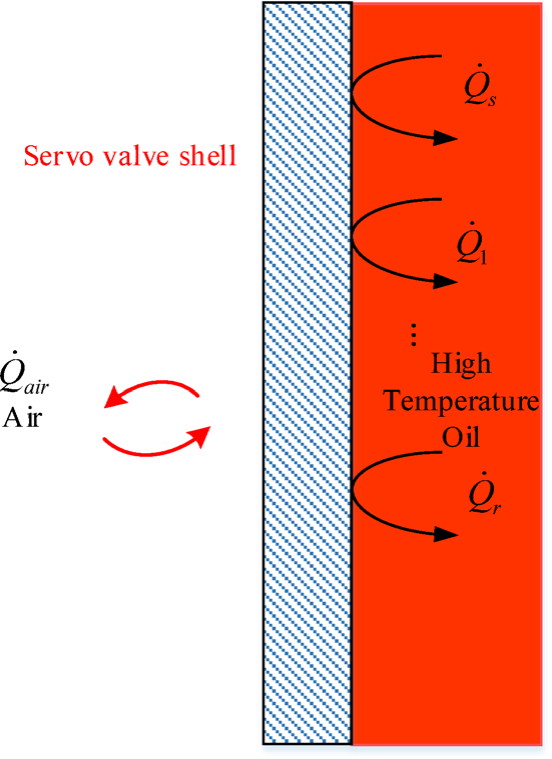
Heat transfer model of the deflector jet servo valve shell.

The heat exchange model between the shell, oil, and air of the deflector jet servo valve is shown in Fig. 6, and the equation for the dynamic temperature ${T_k}$ of the shell of the deflector jet servo valve is

34 $$\frac{{{\text{d}}{T_k}}}{{{\text{d}}t}}=\frac{1}{{{c_{ps}}{m_k}}}[{\dot {Q}_{\text{s}}}+{\dot {Q}_1}+{\dot {Q}_2}+{\dot {Q}_5}+{\dot {Q}_6}+{\dot {Q}_r} - {\dot {Q}_{air}}]$$

where ${c_{ps}}$ is the specific heat capacity of the shell material of the deflector jet servo valve, and ${m_k}$ is the shell mass of the deflector jet servo valve.

### Dynamic temperature profile based on hermodynamic model

The Runge–Kutta method is used to obtain a numerical solution of the thermodynamic mixed ordinary differential equation of the pre-stage of the deflector jet servo valve presented in the previous section. The simulation step is set as 0.005 s, the simulation time is 300 s, and the parameters of the deflector jet servo valve are as described in a previous paper^11^. Initially, each chamber inside the servo valve is filled with oil, the initial temperature is set as the oil supply temperature, and the initial temperature of the shell is set as the ambient temperature. In the calculation, the change time of the set temperature is much longer than the change time of the pressure, and the pressure change rate of the first step affects the temperature change of step *k* in a small $k - 1$ step. Therefore, Eq. (13) simplifies to

35 $${\left. {\frac{{{\text{d}}T}}{{{\text{d}}t}}} \right|_k}=\frac{1}{{{c_p}m}}[{\alpha _p}Tm\nu {\left. {\frac{{{\text{d}}P}}{{{\text{d}}t}}} \right|_{k - 1}}+\dot {Q}+\sum {{{\dot {m}}_{in}}({h_{in}} - h)} ]$$

**Fig. 7:**
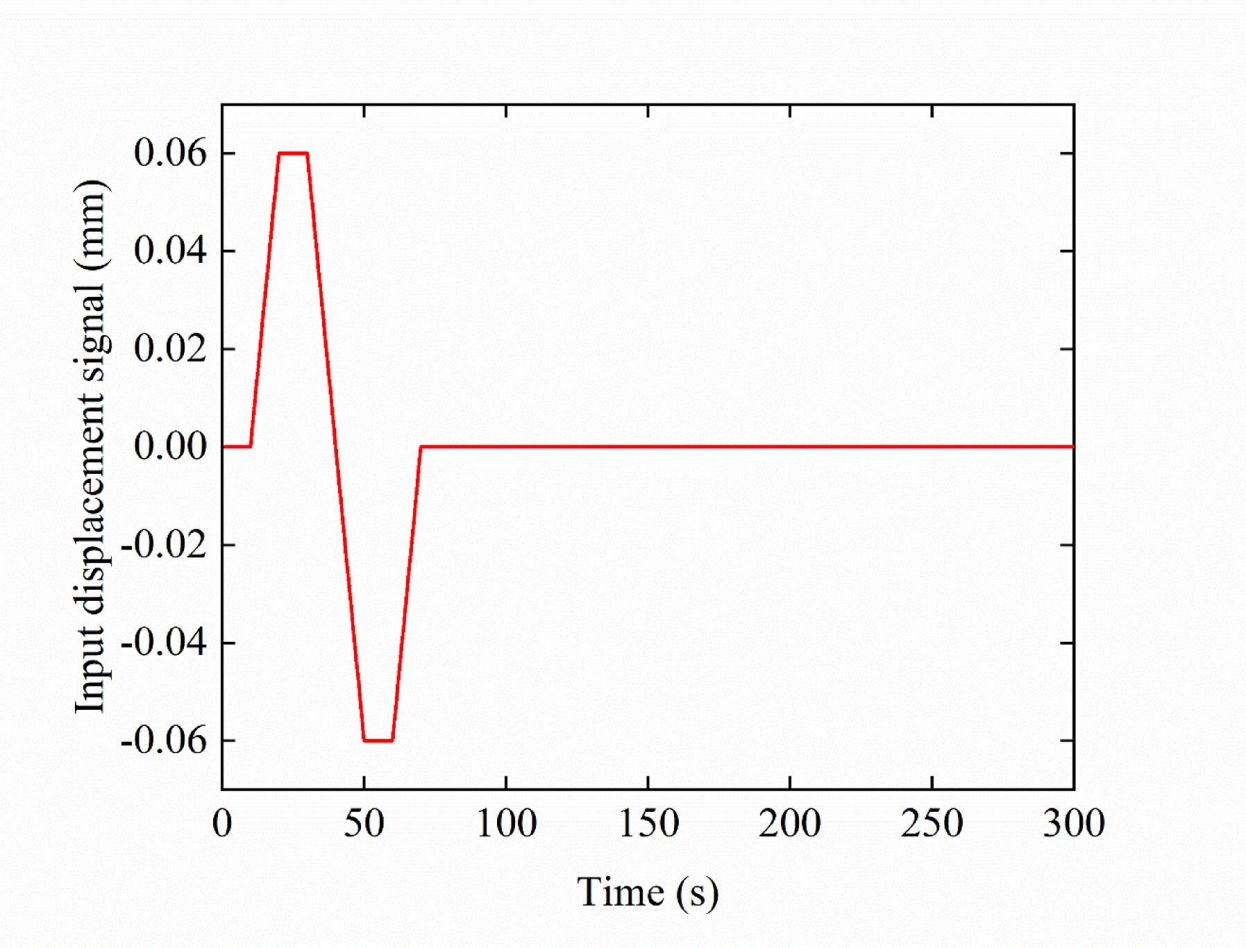
Input displacement signal of the deflector.

In the simulation, the deflector jet servo valve takes the offset position of the pre-stage deflector as input and the temperature and pressure flow rate in each chamber as output. Changes in the input signals over time are shown in Fig. 7. From 0 to 10 s, the deflector remains in the middle position; from 10 to 20 s, the deflector moves by 0.06 mm to the left and remains there for 10 s; from 30 to 50 s, it moves by −0.06 mm in the opposite direction and remains there for 10 s. Finally, it returns to the middle position at 60 s and remains there until the end of the simulation.

**Fig. 8:**
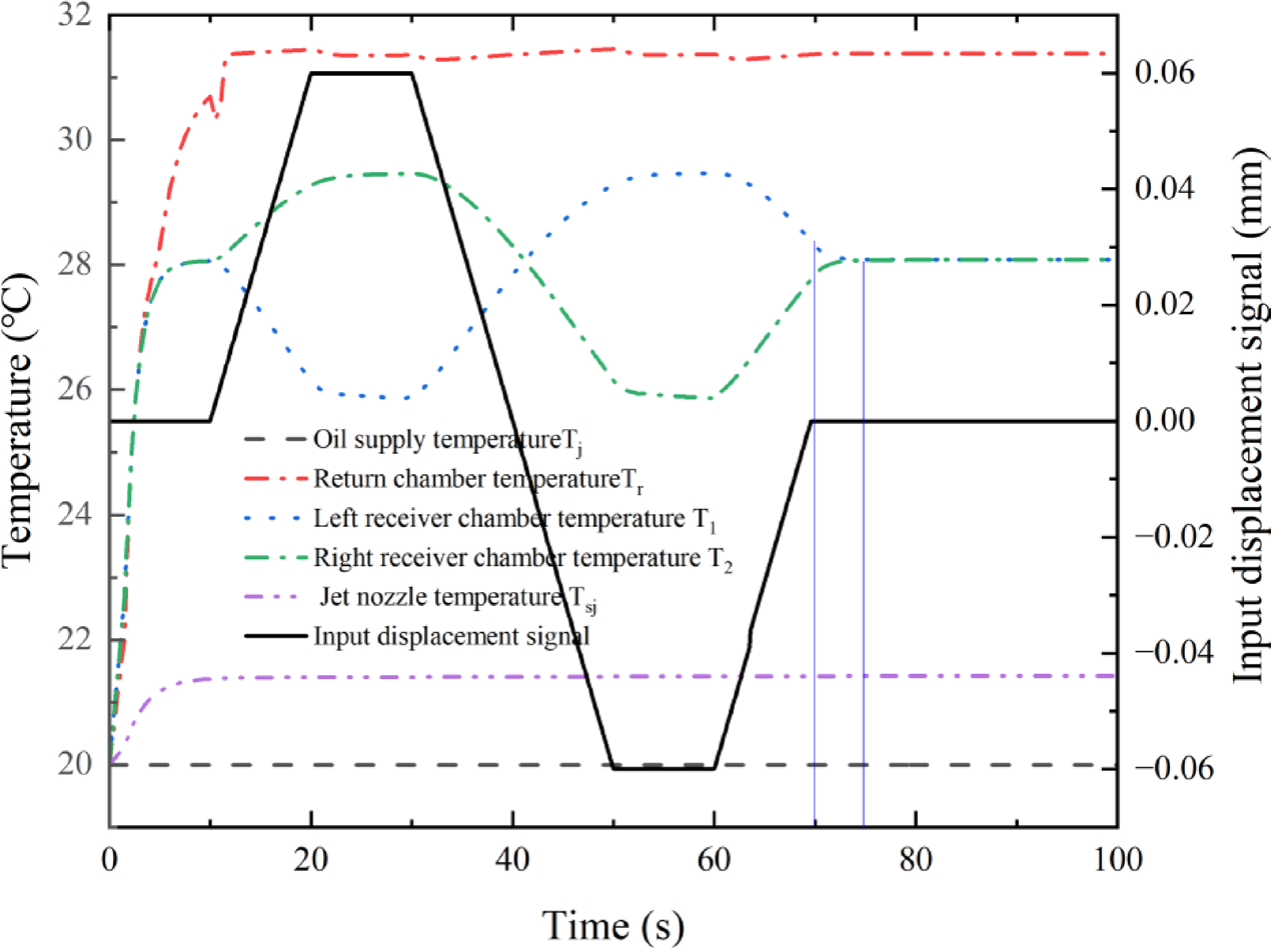
Temperature of each chamber in the deflector jet servo valve.

Under the control of the deflector input displacement signal, the oil temperature of each chamber of the pre-stage changes with the deflection of the deflector position at room temperature (20 ℃), as shown in Fig. 8. In the simulation, the deflector returned to the middle position in 60 s and remained there until the end of the simulation (300 s). Thus, the influence of the input displacement signal of the deflector in the first 100 s on the temperature of each chamber of the pre-stage was observed.

During the period of 0–10 s, the deflector is in the middle position, the deflector jet servo valve starts to supply oil to the oil chamber, the initial jet flow pressure change is small, and the deflector control chamber temperature is 20 ℃. The storage chamber contains more oil, owing to the initial jet flow and secondary jet flow heating oil injection, in addition to the original lower oil temperature of the accumulated oil. At this time, the oil temperature in the control chamber of the deflector and the left and right receiver chambers shows a rising trend, as shown by process ① in Fig. 8. As the deflector gradually moves to the left, the flow area gradually increases, the momentum of the jet received in the left receiver chamber becomes greater than that in the right receiver chamber. Thus, the recovery pressure in the left receiver chamber is greater than that in the right receiver chamber, and the throttle effect of the left receiver chamber gradually increases, with a large amount of oil flowing into the left control chamber mixed as well as considering the oil temperature in the left control chamber. This results in a gradual decrease in the mixing temperature of the left control chamber, as shown by process ⑦ in Fig. 8. Additionally, owing to the large internal volume of the receiver chamber, the flowing oil mixes slowly in the chamber, and the volume of the receiver chamber exhibits a certain lag effect, as shown by process ⑧ in Fig. 8. Similarly, as the deflector moves toward the left, the flow area in regions $A{e_1}$ and $A{e_3}$ reduces, and the pressure loss of the variable throttling device in the right receiver chamber gradually decreases, causing the mixing temperature in the right receiver chamber to gradually increase.

During the period of 20–30 s, the deflector remains at 0.06 mm, the flow in the left receiver chamber reaches its maximum, and the temperature quickly reaches a stable state after the rapid mixing of oil, as shown by process ⑥ in Fig. 8. In contrast, the flow rate of the right receiver chamber is the lowest, the oil mixing speed is low, and the temperature increases slowly and does not fully reach a stable state, as shown by process ⑧ in Fig. 8.

Within 30–50 s, the deflector slowly moves from 0.06 mm to −0.06 mm on the left side, and the movement direction of the deflector is opposite to that in the period of 10–30 s, with the temperature inside each chamber of the pre-stage exhibiting an opposite trend.

The deflector remains at −0.06 mm on the right side between 50 and 60 s and then returns to the middle between 60 and 70 s. The temperature change of the oil mixture in the left and right receiver chambers is significantly delayed by the movement of the deflector, as shown by processes ⑩ and ⑫ in Fig. 8.

The deflector reaches the middle position at 70 s, but the left and right receiver chamber temperatures do not reach equilibrium until 78 s, representing a lag time of approximately 8 s.

**Fig. 9:**
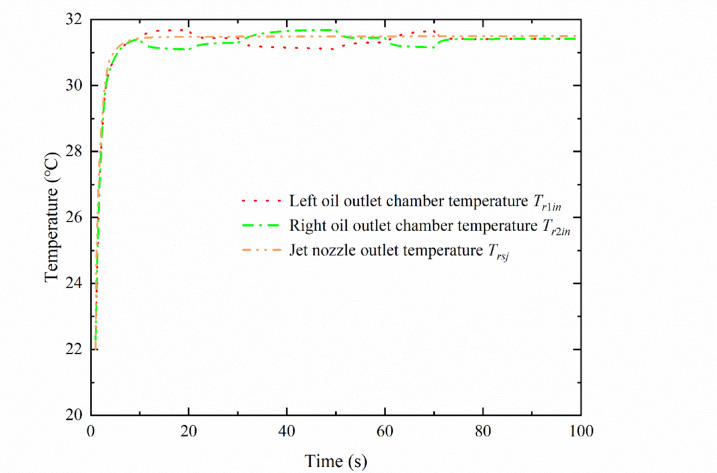
Outlet temperature of the left and right outlet chambers and spool valve.

The temperature of the jet flow nozzle outlet and the temperature of the left and right oil outlet chambers are shown in Fig. 9. During 0–10 s, the oil inlet chamber begins to supply oil. Part of the oil through the primary and secondary jets impacts the receiver platform and is shot into the left and right receiver chambers, while the other part flows back to the tank through the left and right oil outlet chambers. The corresponding maximum temperature is 31.68 ℃. The temperature rise in the oil return chamber is mainly caused by the outflow of the secondary jet flow, as shown by process ② in Fig. 9. During 10–20 s, owing to the volumetric hysteresis effect, the temperature in the left oil outlet chamber increases slowly, as shown by process ④ in Fig. 9. During 20–30 s, the deflector remains at 0.06 mm for 10 s. During this time, the flow and pressure do not change. Because the steady-state temperature difference of the adiabatic throttle element is only related to the pressure difference^25,26^, the temperature of the left oil outlet chamber tends to be in a steady state. Similar temperature fluctuations occur in the oil outlet chamber within 30–50 s, which are mainly a result of the relatively low flow rate of the deflector jet servo valve in the front stage and the volume lag effect in the control chamber.

**Fig. 10:**
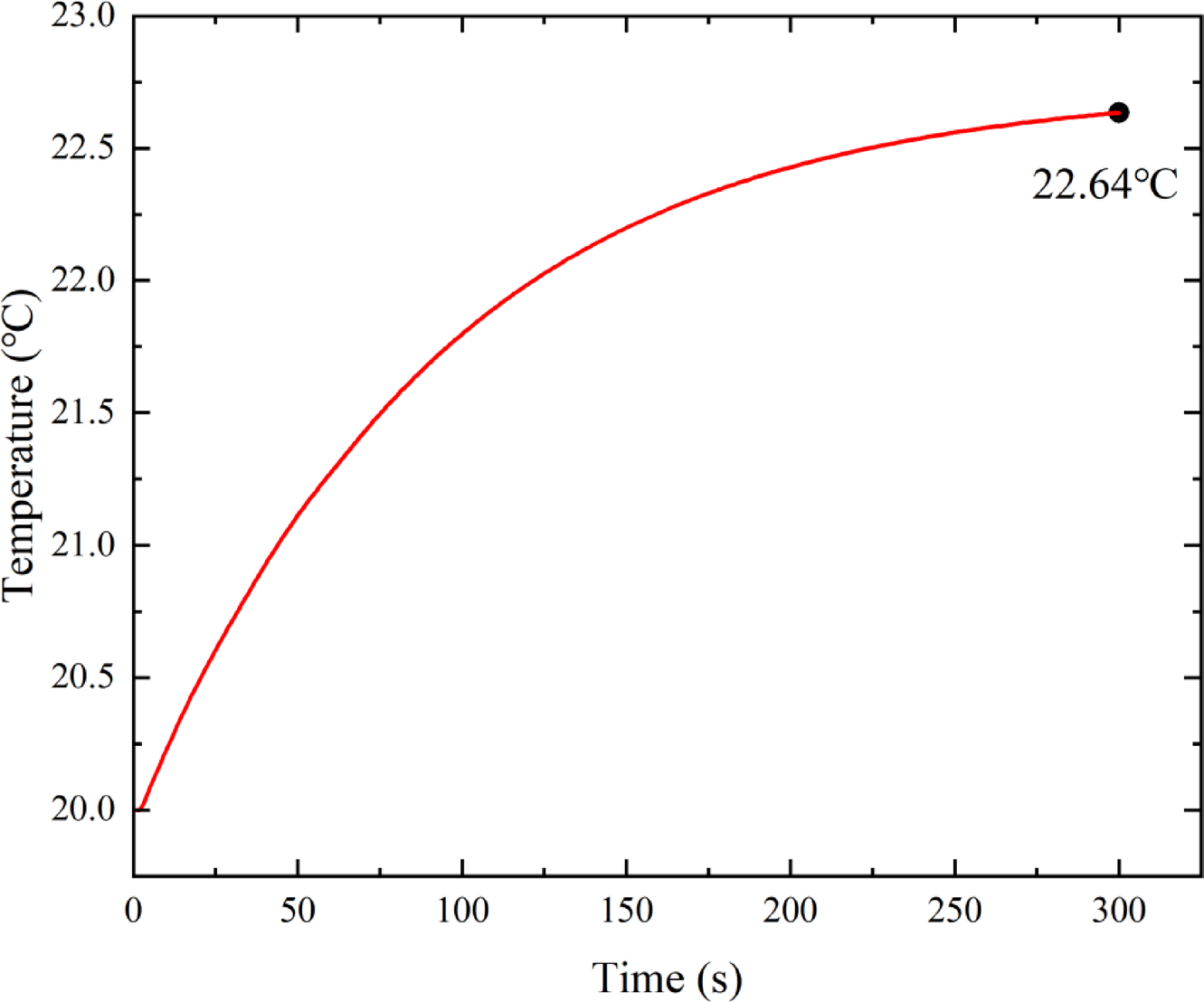
Temperature rise curve of the deflector jet servo valve shell.

Even as the deflector moves to the middle position at 70 s, the numerical simulation continues to calculate the casing temperature of the deflector jet servo valve. The casing of the deflector jet servo valve is used as a heat conduction medium, with a small contact area between the flow channel in the casing and the oil, and no heat dissipation and air convection measures outside the casing. Therefore, the heat flow in and out of the shell is limited, which makes the temperature rise of the deflector jet servo valve shell relatively slow (Fig. 10). Shell temperature is 22.64 ℃ at 300 s and tends to be in an equilibrium state. At this point, the ambient temperature of the oil and the shell reaches steady-state thermal equilibrium. When the temperature cooling effect of the oil source pump station is satisfactory, the temperature rise of the deflector jet servo valve shell is not evident, with an increase of only 2.64 ℃.

## Experimental design and analysis of results

### Temperature measurement method for deflector jet servo valve

**Fig. 11:**
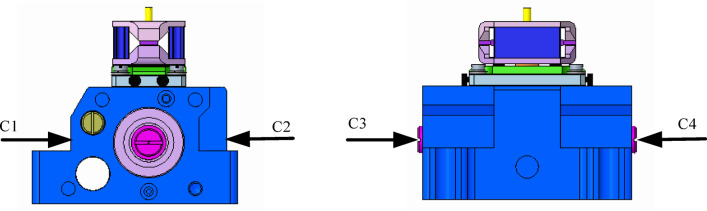
Distribution of temperature measurement points of the deflector jet servo valve.

In this section, thermocouples are used to measure the temperature distribution of the shell of the deflector jet servo valve, and temperature measurement points are set on the front, back, left, and right sides of the shell. The distribution of the temperature measurement points is shown in Fig. 11. To accurately measure the temperature at these points, it is necessary to ensure effective contact between the measuring end of the thermocouple and the measurement point of the deflector jet servo valve. To this end, K-type thermocouples were pasted directly on the corresponding points on the surface of the shell. A dual-channel YET-610 temperature-measuring instrument was used to record data in real time. YET-610 mainly applies the thermocouple temperature measurement principle, with a resolution of 0.01 ℃ and the temperature change curve displayed. It also has a point calibration function, and multi-point calibration can considerably improve its measurement accuracy.

**Fig. 12:**
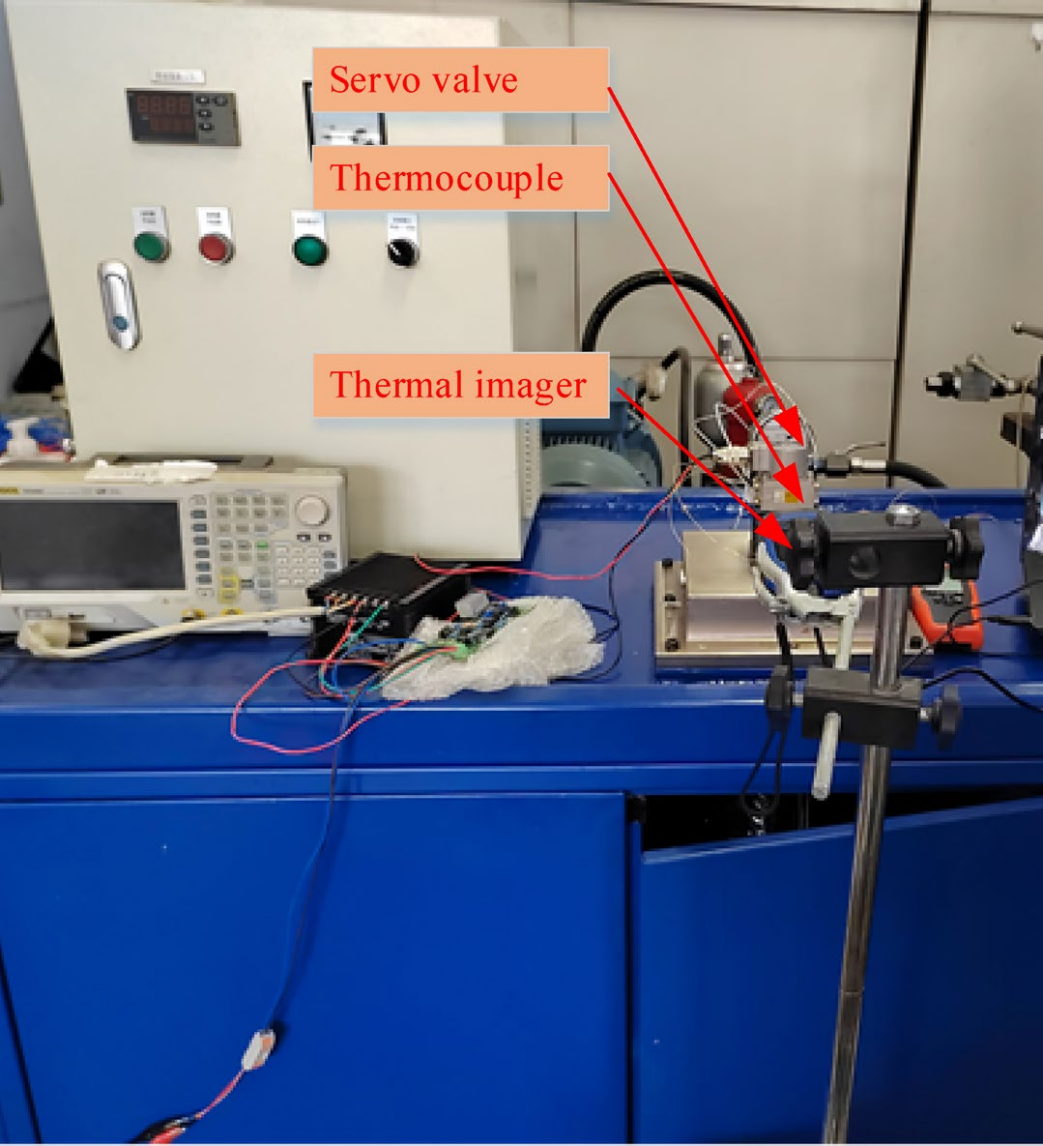
Temperature-measuring device of the deflector jet servo valve.

In experiments focusing on the temperature rise of the deflector jet servo valve, the thermocouple contact temperature measurement method can only measure the temperature distribution with limited temperature measurement points. It cannot obtain the temperature distribution of the deflector jet servo valve shell. Therefore, a handheld infrared thermal imager by Reveal was used as an auxiliary temperature measurement method to measure the temperature distribution of the shell of the deflector jet servo valve during the experiment. This imager generates thermal images and temperature values by detecting the infrared energy radiated from the target and converting it into electrical signals. The accuracy of the thermocouple system is ± 0.5 °C. After accounting for installation errors, the expanded uncertainty is U = ± 0.6 °C (coverage factor k = 2, confidence level 95%). The spatial distribution and extreme temperatures measured by infrared thermal imaging and thermocouples are consistent, with a maximum temperature difference of < 0.5 °C, which is lower than the typical uncertainty of thermal imagers (± 2 °C). The temperature measurement device of the deflector jet servo valve is shown in Fig. 12. The hydraulic oil used for the experiment is 10# aviation hydraulic oil, and the oil temperature characteristics are shown in Table 1^9^.

**Table 1:** Dynamic viscosity value of 10^#^ aviation hydraulic oil at different temperatures.

Temperature(℃)	Dynamic viscosity(Pa·s)
15	0.02
20	0.017
25	0.015
35	0.012
45	0.01
50	0.0085
84	0.006
102	0.004
165	0.002

### Temperature distribution of deflector jet servo valve

**Fig. 13:**
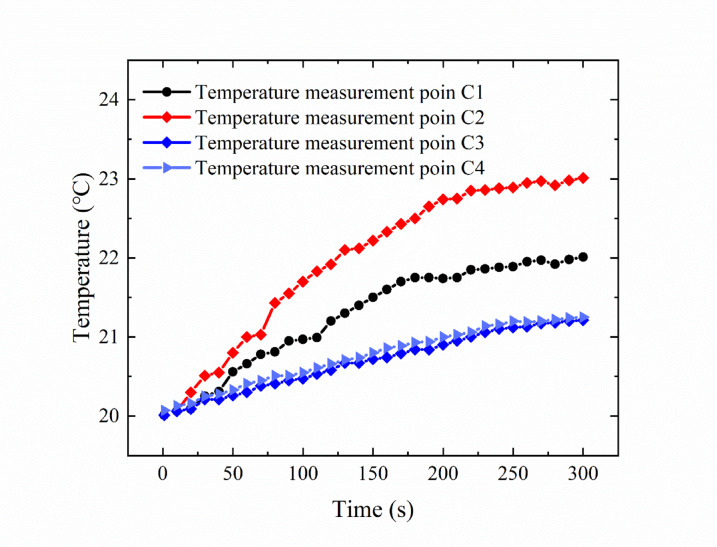
Temperature distribution of the deflector jet servo valve shell measured by thermocouples.

Figure 13 shows the temperature distribution of the shell of the deflector jet servo valve as measured by thermocouples when the oil supply temperature is 20 ℃. Initially, there is little difference in the values recorded at the four points, and shell temperature stabilized at 20 °C. With increasing time, the temperature at each point continuously increases. By comparing the temperature values recorded at the four points, the temperature at points C3 and C4 is lower than that at points C1 and C2. This temperature difference is caused by the shell structure. Points C3 and C4 are located at the edge of the shell. Direct contact between the shell and the outside air causes the lowest temperature to be recorded at the outer edge of the shell of the deflector jet servo valve. Meanwhile, points C1 and C2 are located on both sides of the oil inlet channel and the oil return channel. The temperature here is affected by the temperature of the oil inlet chamber and the temperature of the oil return chamber. These values are higher than those at the shell edge.

Moreover, the temperature recorded at point C2 is higher than that recorded at C1. Point C2 is located on the side of the oil return channel, where the temperature rise rate is the fastest, with the temperature recorded increasing to 23.01 ℃. This is followed by the value recorded at point C1, with a reading of 22.05 ℃. The temperature difference between the two points is approximately 1℃. This is because after the oil passes through the pre-stage and the spool valve chamber, there is a large pressure difference in the throttle port inside the throttle control body. The loss of pressure energy is transformed into viscous dissipation of the oil, generating heat and resulting in a temperature rise in the oil return chamber. Points C3 and C4 are located at the edge of the shell, where the temperature rise is relatively slow. The temperature difference between these points is approximately 0.04 ℃. This is due to the asymmetry of the structure of the deflector jet servo valve, resulting in an uneven heat distribution in the shell. Such inhomogeneity may lead to local deformation, thus affecting the working performance and service life of the deflector jet servo valve.

**Fig. 14:**
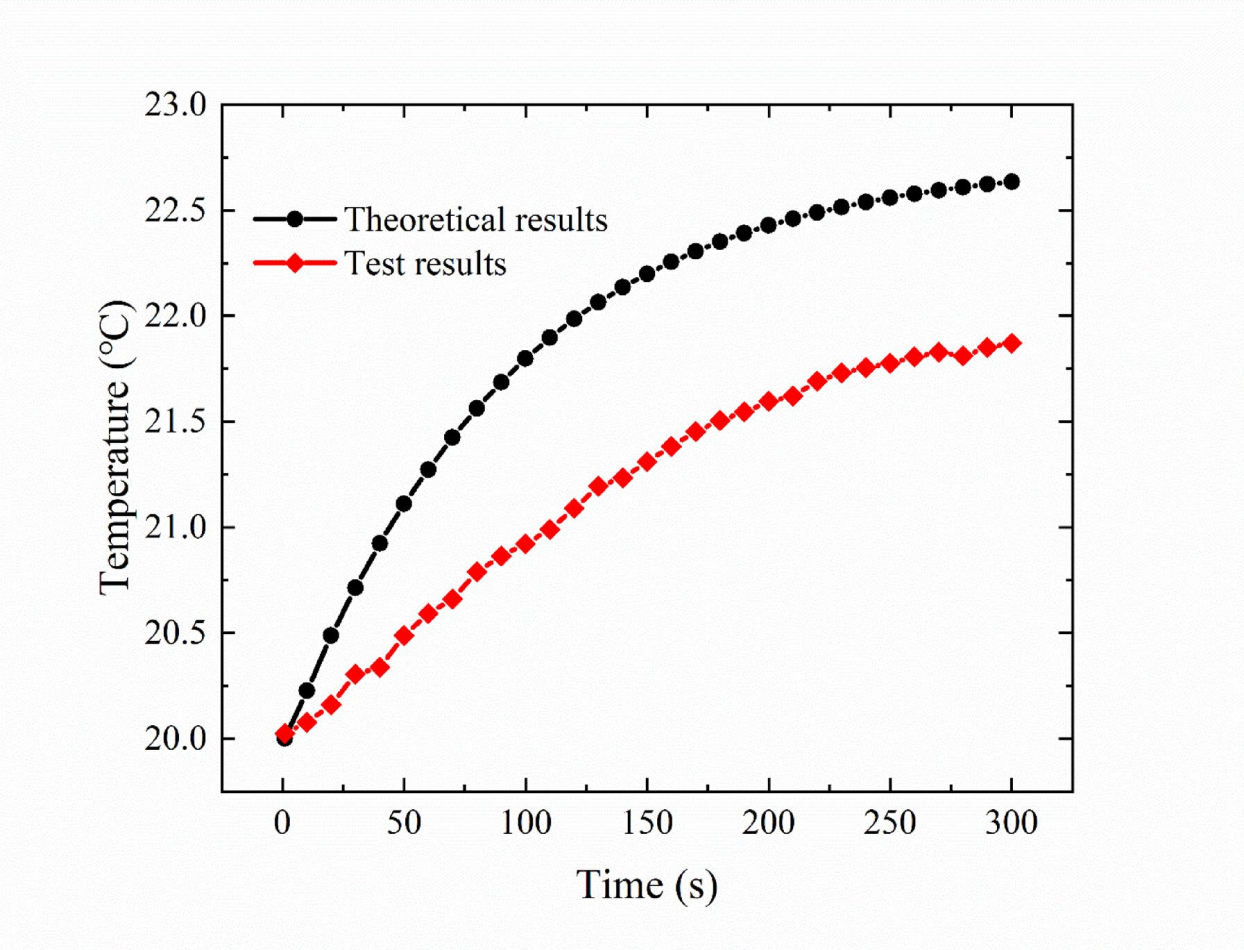
Comparison between the temperature distribution of the deflector jet servo valve shell obtained via experiments and theoretical calculations (with an oil supply temperature of 20 ℃).

Figure 14 compares the average temperature distribution of the deflector jet servo valve shell determined experimentally with that obtained via theoretical calculations when the oil supply temperature is 20 ℃. The results indicate that the experimental results yield lower values than the theoretical analysis results, and the maximum difference is approximately 0.76 ℃. This is because in the theoretical calculations, to simplify the model, the heat conduction process inside the deflector jet servo valve shell is not considered, and internal heat transfer in the shell is considered to be uniform and rapid. Therefore, the experimental results deviate slightly from the theoretical results, but the overall trend is consistent, proving the reliability of the theoretical model.

**Fig. 15:**
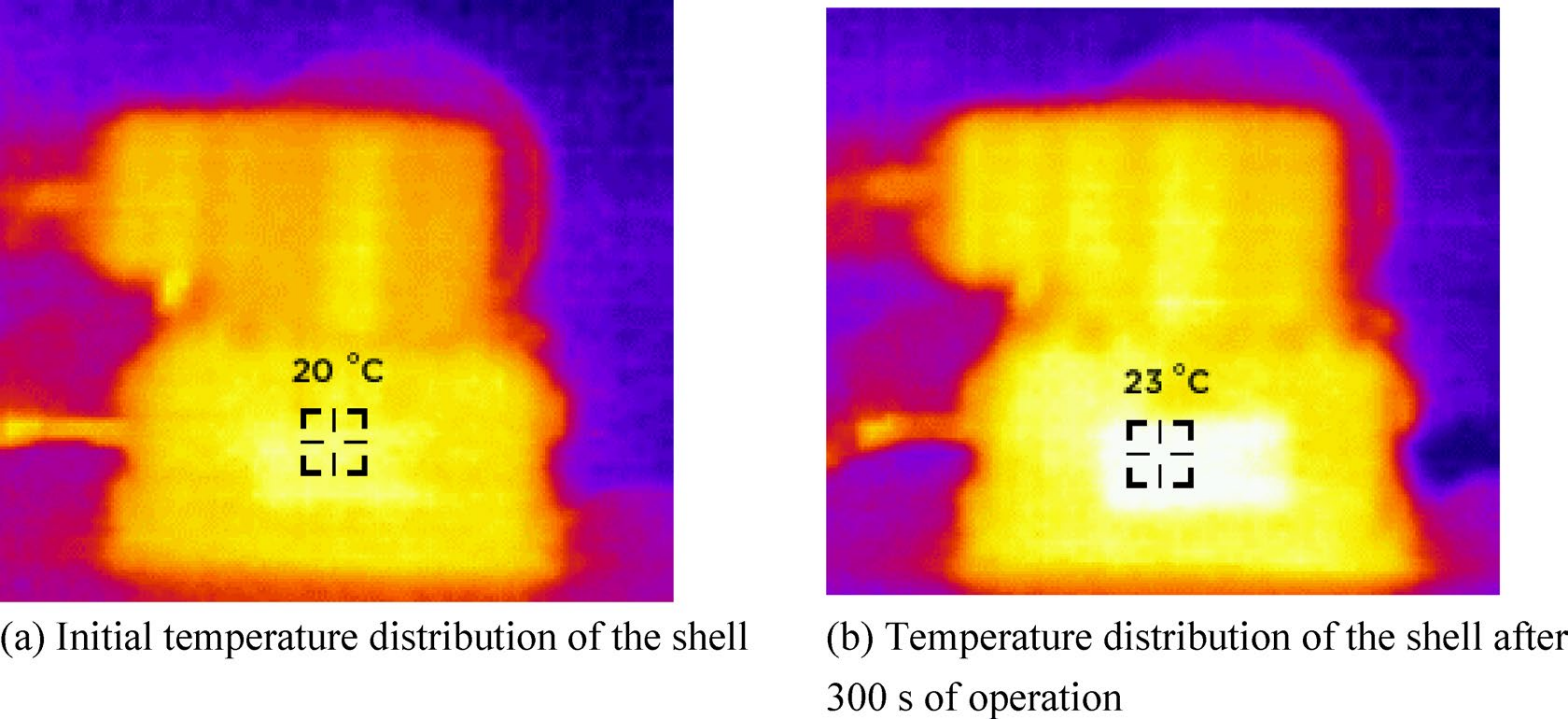
Temperature distribution of the deflector jet servo valve shell obtained using infrared thermography.

Figure 15 shows the temperature distribution in the shell of the deflector jet servo valve obtained using the infrared thermal imager. When the oil supply temperature is 20 °C, the actual temperature measured on the surface of the shell is also 20 °C. The maximum temperature of the shell surface increases to 23 °C after the deflector jet servo valve runs for 300 s, which is almost consistent with the temperature distribution measured by the thermocouples. By comparing with the theoretical calculation results in Fig. 10, the shell temperature gradually increases as the oil temperature rises, which is consistent with the trend of the theoretical calculation results, verifying the correctness of the model. The discrepancy in the theoretical calculation results is primarily caused by thermal diffusion between the servo valve and the air during the experimental process.

### Effect of temperature change on internal leakage

A high-temperature characteristic test rig was used to measure the internal leakage of the deflector jet servo valve. The oil supply pressure was set to 21 MPa, and the flow rate of the oil return chamber was measured when the servo valve was in the median working condition. The test results after the first 180 s were recorded several times and the average data were considered. Table 2 shows the leakage flow of the oil chamber under different operating times of the servo valve at no load. The results indicates that the oil temperature increases rapidly within 90 s, and internal leakage of the servo valve gradually increases, with its power loss continuously increasing. With increasing running time, the oil temperature gradually tends to a stable state, with almost no change in internal leakage. The leakage increased by 13.5% within 180 s. The flow rate increased by 0.113 L/min, the power loss is 24 W.

**Table 2:** Internal leakage flow rate of the deflector jet servo valve.

Time (s)	Mean leakage (L/min)	SD (L/min)	SEM (L/min)	95% CI (L/min)
5	0.8346	0.0055	0.0025	[0.827, 0.842]
10	0.9200	0.0037	0.0017	[0.915, 0.925]
30	0.9360	0.0030	0.0013	[0.932, 0.940]
60	0.9420	0.0022	0.0010	[0.939, 0.945]
90	0.9450	0.0016	0.0007	[0.943, 0.947]
120	0.9472	0.0008	0.0004	[0.946, 0.948]
150	0.9450	0.0022	0.0010	[0.942, 0.948]

**Fig. 16:**
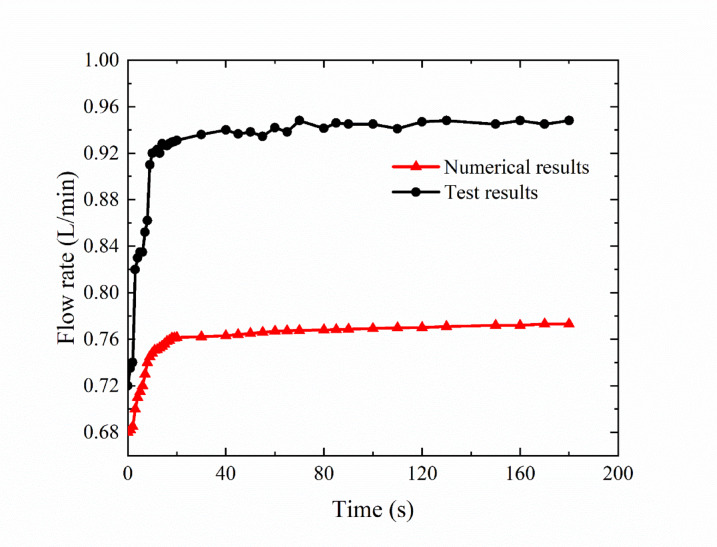
Comparison of test and numerical simulation results of the leakage flow rate of the deflector jet servo valve.

Figure 16 compares the leakage test results and the numerical simulation results of the deflector jet servo valve. The trend of internal leakage flow obtained by the test and the numerical simulation is consistent, with the leakage flow gradually increasing with time. However, the test results exhibit certain large values and fluctuations, which may have been caused by the structural asymmetry phenomenon in the machining and assembly process of the servo valve.

With increasing running time, the oil temperature rises rapidly, and the change in temperature may cause the zero position of the deflector jet servo valve to change, resulting in fluctuations in the test data. The influence of temperature changes on the control accuracy of the servo valve is verified. However, the leakage measured in the test analysis includes not only the leakage in the pre-stage flow field but also the leakage flow between the valve spool and valve sleeve of the power stage, which may result in higher values in the test data. Temperature-induced leakage may mean that maintenance is needed more frequently.

## Conclusion

Based on heat transfer and thermodynamics theory, a thermodynamic model of the pre-stage of a deflector jet servo valve was established using the control body method. With the displacement of the deflector as input, the mixed differential equation is solved numerically, and the temperature rise of each chamber inside the servo valve is predicted. The accuracy of the thermodynamic model through experimentation is verified. The following conclusions are drawn:

(1) The mechanism through which the oil flows inside the deflector jet servo valve is simplified into several volumetric and damping control bodies. A pre-stage thermodynamic model of the deflector jet servo valve is established.

(2) With the displacement of the deflector as the input, the pre-stage thermodynamic model of the deflector jet servo valve is solved, and the temperature change versus time curve of each chamber and shell in the servo valve was predicted. When the deflector returned to the middle position after 70 s, because of the volume effect of the pre-stage, the temperature in the left and right receiver chambers did not reach an equilibrium state until 78 s, representing a lag time of approximately 8 s.

This study improves the thermal modeling of servo valves. The experimental-model correlation, industrial reliability standards, and control implications will be studied further in the future.

## Data Availability

The data supporting the findings of this study are available within the article and its supplementary material. Additional data are available from the corresponding author upon reasonable request.
